# Supplementary material for: Pupil size reveals the perceptual quality and effortless nature of synesthesia
Source: eLife. 2026 Aug 4;15:RP110390. doi: 10.7554/eLife.110390 (PMC13436957; doi:10.7554/eLife.110390)
Supplement: Peer review history

# Editor report: eLife Assessment

**DOI:** 10.7554/eLife.110390.4.sa0

**Authors:** Joshua I Gold

**Affiliations:** University of Pennsylvania United States

**Keywords:** Convincing, Valuable

This study used pupillometry to provide an objective assessment of a form of synesthesia in which people see additional color when reading numbers. It provides **convincing** evidence that subjective color ratings are matched by changes in pupil size that recapitulate brightness-mediated changes when exposed to the real color. The work provides a **valuable** contribution to the literature on both synesthetic perception and the use of pupillometry to probe perception and related psychological processes.

# Referee report: Reviewer #1 (Public review)

**DOI:** 10.7554/eLife.110390.4.sa1

[Editors' note: this version has been assessed by the Reviewing Editor without further input from the original reviewers. The authors have addressed the comments raised in the previous round of review.]

Summary:

Knowing that small pupil-size variations accompany brightness variations (even when these are illusory), the authors asked whether pupil constrictions would accompany the synesthetic perception of a brighter color (compared with a darker one), induced by the presentation of a black-white character. This grapheme-colour synesthesia is only experienced by few participants, sixteen of whom were enrolled in this study. The results reliably showed that a relative pupil constriction would "betray" the perception of a brighter color in these participants, while no such effect would be observed in control participants who were asked to report a color in association with each grapheme, even though they did not perceive any.

Strengths:

The main strength of the study lays in its combination of psychophysics (brightness ratings) and pupillometry, which allowed for showing clear-cut results.

Impact:

This work is likely to improve our understanding of synesthesia, providing a new tool to quantify the subjective sensations; an interesting potential extension would be using pupillometry for tracking changes over time of the synesthetic experiences, opening up the possibility to evaluate the importance of learning for this peculiar experience.

# Referee report: Reviewer #2 (Public review)

**DOI:** 10.7554/eLife.110390.4.sa2

Synesthesia is a neurological condition where stimulation of one sensory channel leads to involuntary, automatic, and consistent experience of another, unrelated percept. For example, Sir Francis Galton (1880, Nature) famously described the robust tendency of some individual (synesthetes) to associate numerals with a distinct color. Ever since, synesthesia keeps attracting a broad interest in the cognitive neurosciences in light of its implications for the study of domains such as perception, consciousness, and brain connectivity, among others.

Strauch, Leenaars, and Rouw measured pupil size in a group of 16 grapheme-color synesthetes and two matched control groups. The participants were presented with gray digits - that is, visual stimuli having identical physical properties in terms of brightness. Each participant subsequently rated the corresponding evoked color and brightness: unlike controls, synesthetes did so in a very consistent and reliable fashion. Accordingly, this was also shown in their pupils: despite the same objective luminance, digits associated with brighter percepts caused their pupils to constrict and digits associated with darker percepts caused their pupils to dilate more than controls. These results highlight how crossmodal correspondences are deeply rooted in synesthetes, and puts forward pupillometry as a particularly appealing biomarker for some phenomenological experience (at least those grounded in "brightness").

Further strengths of the technique are its temporal resolution and its responsiveness to several constructs. Across several tasks, the authors show for example that responses to synesthetic light are somewhat slower than responses to real light (i.e., they are likely mediated), but at the same time faster than responses to mental imagery. The role of mental imagery can also be reasonably dismissed when considering the second feature of pupil size: its responsiveness to mental effort and cognitive load. The pupils tend to dilate with demanding, challenging tasks, and this was the case when control participants were asked to report the color of a digit for which they did not consistently experience a synesthetic association. The same task was, instead, seemingly effortless for synesthetes, again speaking in favor of the automaticity of number-color correspondences in their case.

Overall, the findings by Strauch, Leenaars, and Rouw are highly significant for the field and likely to be impactful. The strength of their evidence, when accounting for the relatively small sample size and the inherent variability of both phenomenology (color perception and subjective reporting) and physiology (pupil size), is adequate and sufficiently convincing.

# Author comment: Author response

**DOI:** 10.7554/eLife.110390.4.sa3

**Authors:** Christoph Strauch, Casper Leenaars, Romke Rouw

**Affiliations:** Experimental Psychology, Helmholtz Institute, Utrecht University Utrecht Netherlands; Experimental Psychology, Helmholtz Institute, Utrecht University Utrecht Netherlands; University of Amsterdam Amsterdam Netherlands

The following is the authors’ response to the previous reviews

**Public Reviews:**

**Reviewer #1 (Public review):**

The pupil traces in Figure3 (main results) are heavily pre-processed (per-participant demeaned), loosing any feature besides the effect of interest. As I argued in my first review, I worry that this format gives unrealistic expectations about the effect (the perception of dark/bright colors do not generate a net dilation/constriction of the pupil; perception-related modulations of pupil size are always relative and generally small compared to the numerous other effects registered in pupil size; these include a pupil dilation that is more prominent in the controls and that gets analyzed later on in the manuscript; I do not think that eliminating one of the effects of interests from a main results figure helps the reader understand the results). In the revised manuscript, the authors addressed this concern by adding a Supplementary Figure 4, where a more complete representation of the results is shown (traces from individual trials are baseline corrected and averaged, resulting in more informative timecourses). I would strongly recommend that Supplementary Figure 4 is brought to the main text (Figure 3 could be presented in Supplementary).

We agree that it is important to counter unrealistic interpretations of the effect. However, figures in the main article are the ones that are depicting the effects. Instead, it seems that additional clarification on these effects is needed. First and foremost, Figure 3 in the main manuscript visualizes the core effect: pupil size reveals that synesthesia is a sensory process and the phenomenology of the synesthetic experience can be measured physiologically. Secondly, this allows to advance synesthesia (and phenomenology) research as a new and powerful method.

No doubt, our effect is relative in nature (as almost any pupillometry, fmri, eeg effect etc.). Including variation that is unrelated to the effect would increase rather than decrease confusion, as individual differences (i.e., how the pupil of an individual responds irrespective of the synesthetic experience) are unmeaningful to the question we set out to answer. Individual variations in pupil response shape irrespective of synesthetic color brightness are removed in Figure 3 but still present in Supplementary Figure 4. Thus, Figure 3 is better suited to illustrate our core effect than Supplementary Figure 4, as individual average responses (illustrated on the right) cannot be meaningfully related to the core effect anymore, only the difference can be.

At the same time, the reviewer is correct that this may, not so much among researchers as among a general audience, create the expectation that the pupil will always net dilate when experiencing a dark synesthetic percept. This is clearly not the case, but only over its counterfactual (i.e., not seeing that dark synesthetic percept). We now counter such an unrealistic expectation:

“Note that the effects here are visualized as counterfactuals. So while the pupil dilated for dark relative to bright experienced colors in synesthetes, this does not mean that the pupil net dilates and constricts to dark and bright experienced colors relative to baseline, but only relative to the counterfactual (see Supplementary Figure 4 for net pupil size changes).”

We updated the caption of Supplementary Figure 4 as follows:

"Supplementary Figure 4: Pupil size change to graphemes, split by 0.5 reported color lightness (dark gray = low lightness; light gray = high lightness) without demeaning (i.e., removing the average pupil response shape in the 4s stimulus interval per individual irrespective of brightness perception). (…)"

Responses to physical brightness modulations were only measured in the synesthethes group, not in controls. The authors point out that pupillary light responses have been thoroughly characterized in previous studies, and conclude that synesthethes' responses were in line with the expectations both in terms of amplitude and latency. However, as we are not dealing with standardized measurements, subtle differences in pupil reactivity across the two populations remain a possibility. I recommend that this possibility is mentioned in the discussion.

We agree with the reviewer, if there were any differences in the PLR between the two groups, they must be minor given that the responses follow those reported in the literature so closely. Yet, subtle differences cannot be ruled out fully unless tested and it doesn’t hurt mentioning this in the discussion, which we now do as follows:

Finally, pupil light responses in Block 2 were only assessed in synesthetes. While these closely match such of control populations [50,51], subtle between-group differences cannot be excluded and could ideally be assessed in future and replication work.

**Reviewer #2 (Public review):**

Synesthesia is a neurological condition where stimulation of one sensory channel leads to involuntary, automatic, and consistent experience of another, unrelated percept. For example, Sir Francis Galton (1880, Nature) famously described the robust tendency of some individual (synesthetes) to associate numerals with a distinct color. Ever since, synesthesia keeps attracting a broad interest in the cognitive neurosciences in light of its implications for the study of domains such as perception, consciousness, and brain connectivity, among others.

Strauch, Leenaars, and Rouw measured pupil size in a group of 16 grapheme-color synesthetes and two matched control groups. The participants were presented with gray digits - that is, visual stimuli having identical physical properties in terms of brightness. Each participant subsequently rated the corresponding evoked color and brightness: unlike controls, synesthetes did so in a very consistent and reliable fashion. Accordingly, this was also shown in their pupils: despite the same objective luminance, digits associated with brighter percepts caused their pupils to constrict and digits associated with darker percepts caused their pupils to dilate more than controls. These results highlight how crossmodal correspondences are deeply rooted in synesthetes, and puts forward pupillometry as a particularly appealing biomarker for some phenomenological experience (at least those grounded in "brightness").

Further strengths of the technique are its temporal resolution and its responsiveness to several constructs. Across several tasks, the authors show for example that responses to synesthetic light are somewhat slower than responses to real light (i.e., they are likely mediated), but at the same time faster than responses to mental imagery. The role of mental imagery can also be reasonably dismissed when considering the second feature of pupil size: its responsiveness to mental effort and cognitive load. The pupils tend to dilate with demanding, challenging tasks, and this was the case when control participants were asked to report the color of a digit for which they did not consistently experience a synesthetic association. The same task was, instead, seemingly effortless for synesthetes, again speaking in favor of the automaticity of number-color correspondences in their case.

Overall, the findings by Strauch, Leenaars, and Rouw are highly significant for the field and likely to be impactful. The strength of their evidence, when accounting for the relatively small sample size and the inherent variability of both phenomenology (color perception and subjective reporting) and physiology (pupil size), is adequate and sufficiently convincing.

Comments on revisions:

I thank the authors for addressing all my comments in a satisfactory way. I think that the paper has improved, especially in terms of transparency of the reporting and clarity of the results.

We thank R1, R2, and R3 for their very useful input to improve our manuscript.
